# Manganese-Doped Cesium Lead Halide Perovskite PMMA Composite Fibrous Membranes by Electrospinning for Photocatalytic and LED Applications

**DOI:** 10.1021/acsomega.5c06459

**Published:** 2025-09-25

**Authors:** Yujie Cheng, Yuang Ji, Donghai Lin, Wan Y. Shih, Wei-Heng Shih

**Affiliations:** 1 School of Energy and Materials, Shanghai Thermophysical Properties Big Data Professional Technical Service Platform, Shanghai Engineering Research Center of Advanced Thermal Functional Materials, Shanghai Key Laboratory of Engineering Materials Application and Evaluation, 74598 Shanghai Polytechnic University, Shanghai 201209, China; 2 School of Biomedical Engineering, Science, and Health Systems, Drexel University, Philadelphia 19104, United States; 3 Department of Materials Science and Engineering, 6527 Drexel University, Philadelphia 19104, United States

## Abstract

Motivated by the need to reduce toxic organic dyes in wastewaters, we studied the preparation of different amounts of Mn-doped cesium lead halide perovskite in PMMA matrix (CsPb_*x*_Mn_1–*x*_Cl_*y*_Br_3–*y*_@PMMA) composite fibrous membranes (CFM) as a photocatalyst to degrade dyes via a one-step in situ electrospinning method. The composite fibrous membranes exhibit excellent environmental stability, attributed to the protective three-dimensional network structure of the electrospun fibers, which shields the internal perovskite nanocrystals from direct contact with water and light. Structural characterization by X-ray diffraction (XRD) reveals that Mn^2+^ substitution induces a phase transformation from cubic CsPbBr_3_ to tetragonal CsPbCl_3_. The optimal Mn/Pb ratio of 2.5:1 achieves excellent photocatalytic activity, degrading 94.7% of methyl orange (MO) within 90 min under Xe lamp irradiation with a rate constant of 0.031 min^–1^, 4.6 times higher than that of the undoped sample. In addition, a single monochromatic CFM with a blue LED light source can be combined to construct a white LED (WLED) with good chromaticity coordinates (0.3267, 0.3298) and color temperature of 5775 K. This work highlights the great potential of Mn-doped perovskite composite fibrous membranes for practical photocatalytic and LED applications.

## Graphical abstract

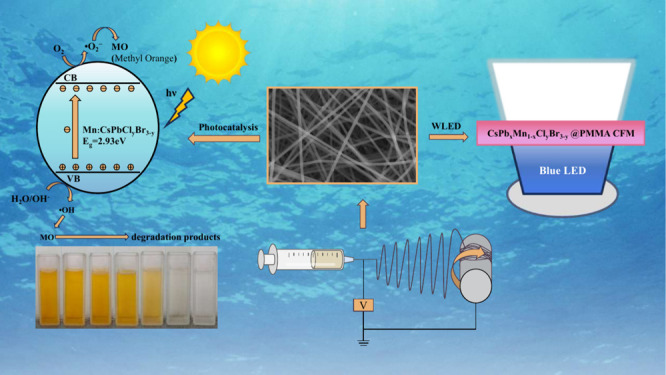

## 1 Introduction

The wastewater discharged from many industrial activities often contains toxic organic dyes, which pose significant threats to the environment and human health. Photocatalytic degradation is an effective method for treating these dyes, as it breaks them down into nontoxic or low toxicity substances, thereby improving water quality.

All-inorganic cesium lead halide perovskite materials (CsPbX_3_, X is halogen) exhibit excellent optoelectronic properties, such as high luminescence quantum yield, narrow emission line widths, adjustable emission wavelength, and high carrier mobility.^1,2^ These properties have made them widely applicable in light-emitting diodes (LEDs),^3–5^ photodetectors,^6^ and solar cells.^7,8^ In recent years, they have also been explored as photocatalysts.^9,10^ However, lead halide perovskite materials are prone to degradation under ambient conditions, such as humidity and light, which reduce their catalytic performance and limit their application in photocatalytic reactions. Their instability in aqueous environments is especially severe for the treatment of dyes in wastewater. Additionally, if cesium lead halide perovskite particles are directly used for photocatalysis, residual lead (Pb) may remain during recovery, posing secondary water pollution risks. Therefore, enhancing their stability and photocatalytic efficiency while mitigating the toxicity of Pb is urgently needed. Various strategies have been proposed to improve the stability of perovskite, including doping,^11,12^ coating,^13^ ligand modification.^14^

Many photocatalysts are in powder form,^15–17^ which, despite their high performance, suffer from common disadvantages, such as easy agglomeration, reduced catalytic activity, and difficulty in separation and recovery from water.^18^ One effective solution is to immobilize powder photocatalyst on a substrate, such as by mixing photocatalyst with polymers using coating^19^ or sol–gel method^20,21^ to create composite films. The composite films have been applied in a variety of areas^18,19^ like sewage treatment, food preservation, and hydrogen evolution reactions. Compared with powder photocatalysts, composite films offer better recovery rates, reducing the toxicity associated with powder photocatalysts. However, composite film photocatalysts may also face challenges, such as uneven dispersion or surface photocatalyst detachment, which can significantly reduce photocatalytic efficiency.^18^ Electrospinning is a versatile technique and has been widely used in photocatalysis.^22^ It is compatible with many polymers and allows for the selection of suitable carriers, ensuring their stability and preventing degradation under light. Electrospun membranes benefit from their large specific surface area and high porosity, which provide more available reaction sites and enhance the efficiency of photogenerated carriers.^10,22^ A high contact angle helps prevent water penetration into the membranes, while uniform catalyst distribution avoids aggregation. Moreover, composite membranes are easier to recycle. Poly­(methyl methacrylate) (PMMA) is chosen as a suitable carrier due to its excellent stability, light resistance, and heat resistance.^9^

In this study, a one-step electrospinning method was employed to synthesize manganese (Mn)-doped cesium lead halide perovskite nanocrystals in situ on PMMA fibers. Mn doping has been shown to increase the lifetime of photogenerated charge carriers, improve their separation efficiency, and localize holes and electrons around Mn, thereby slowing down recombination^16,23^ and enhancing catalytic activity. Moreover, B-site doping reduces the lead (Pb) content and the toxicity of Pb-based perovskite. Dispersing perovskite nanocrystals on membranes minimizes the nanoparticle aggregation and photogenerated hole electron pair recombination. Furthermore, the one-step in situ synthesis method is easier to operate than the traditional two-step method (synthesizing nanocrystals first and then immobilizing them on polymers). Experimental results demonstrate that Mn-doped cesium lead halide perovskite produced by electrospinning exhibits excellent environmental stability. In addition, Mn doping significantly enhances the photocatalytic degradation of organic dyes by cesium lead halide perovskite materials.

Furthermore, due to the presence of new emission peaks caused by Mn doping, we studied the application of a Mn-doped CsPb_*x*_Mn_1–*x*_Cl_*y*_Br_3–*y*_@PMMA composite fibrous membrane as white LED (WLED). There have been several studies of WLED using Mn-doped halide perovskites. In general, WLEDs were prepared by selecting films of different colors according to the different luminescent properties of Mn-doped materials. Hou et al. used Mn doping to increase the photoluminescence of CsPb­(Cl/Br)_3_ blue emission and then stacked red and green films on blue LEDs to create WLEDs,^24^ a common method used in many studies. Chen et al. used green CsPbBr_3_@SiO_2_ and orange Mn:CsPb­(Cl/Br)_3_@SiO_2_ composites as a color shift to construct WLEDs with good optical properties and stability.^25^ Shi et al. dispersed CsPb­(Cl/Br) _3_, CsPbBr_3_, and Mn:Cs_4_PbCl_6_ NC in a toluene solution of polystyrene to make thin films, respectively, and then prepared three-layer WLEDs.^26^ In addition to cesium lead halide perovskite materials, Mn doping has been similarly investigated in other materials. Huang et al. fabricated WLEDs on 360 nm InGaN chips with a combination of the blue phosphor BaMgAl_10_O_17_:Eu^2+^ and the green phosphor BaSrSiO_4_:Eu^2+^ and the orange phosphor CsCdCl_3_:Mn^2+^.^27^ As can be seen from previous studies, to obtain white luminescence, most of them stacked at least two films of different colors. In contrast, we found that a monochromatic Mn-doped CsPb_*x*_Mn_1–*x*_Cl_*y*_Br_3–*y*_@PMMA composite fiber membrane can be combined with a blue LED to produce a WLED. WLEDs made of monochromatic film are easier to prepare than multicolor ones. Also, it is easier to adjust the color by changing the thickness of the film without having to consider the matching between colors in the case of multicolor films. In addition, using the monochromatic film is more reproducible, and the color consistency of the prepared WLEDs is high with less error.

## 2 Experimental Section

### 2.1 Materials

The following materials were used in this study: cesium bromide (CsBr, 99.9%), lead bromide (PbBr_2_, 99%), manganese­(II) chloride anhydrous (MnCl_2_, 99%), oleic acid (OA, C_18_H_34_O_2_, 90%), poly­(methyl methacrylate) (PMMA, AR), oleylamine (OLA, C_8_H_19_N, 90%), *N*,*N*-dimethylformamide (DMF, 99.8%), methyl orange (MO, C_14_H_14_N_3_SO_3_Na, 95%), isopropanol (IPA, 99.9%), and *p*-benzoquinone (BQ, 99%). All materials were purchased from Shanghai Titan Scientific Co., Ltd., and used as received without further purification.

### 2.2 Sample Preparation

#### 2.2.1 CsPb_*x*_Mn_1–*x*_Cl_*y*_Br_3–*y*_@PMMA Precursor

0.2 mmol of CsBr, 0.2 mmol of PbBr_2_, and a corresponding molar amount of MnCl_2_ were added to a glass bottle containing 5 mL of DMF solution. The solution was stirred at 30 °C until it becomes clear and transparent. Subsequently, 0.4 mL OA and 0.1 mL OLA were added under continuous stirring for 30 min. Finally, 1.5 g (30 wt % relative to the total DMF solution) of PMMA was added and the mixture was stirred for 24 h to obtain CsPb_*x*_Mn_1–*x*_Cl_*y*_Br_3–*y*_@PMMA precursor.^28^

#### 2.2.2 CsPb_*x*_Mn_1–*x*_Cl_*y*_Br_3–*y*_@PMMA Electrospun Fibrous Membranes

A 2 mL aliquot of CsPb_*x*_Mn_1–*x*_Cl_*y*_Br_3–*y*_@PMMA precursor solution was loaded into a syringe and mounted onto an electrospinning machine. The positive electrode was connected to the metal needle of the syringe, while the negative electrode was connected to a rotating receiving drum, establishing a high-voltage electrostatic field between the two electrodes. The electrospinning conditions were set as follows: voltage of 15 kV, receiving distance of 15 cm, drum speed of 500 rpm, ambient temperature of 35 °C, humidity of 45%, and feed rate of 0.2 mL/h. The electrospinning process was carried out for 2 h under these conditions. When the precursor solution was extruded from the syringe, the high-voltage electric field induced the formation of a Taylor cone at the needle tip. As the solvent (DMF) evaporated, the precursor solution became supersaturated, promoting the nucleation and growth of the CsPb_*x*_Mn_1–*x*_Cl_*y*_Br_3–*y*_@PMMA nanocrystals. Simultaneously, the electrospun fibers were formed and collected on the rotating drum. After electrospinning, the fibrous membrane was dried in an oven at 50 °C for 4 h to remove residual solvent and obtain the final CsPb_*x*_Mn_1–*x*_Cl_*y*_Br_3–*y*_@PMMA electrospun fibrous membranes.

#### 2.2.3 WLED Fabrication

CsPb_*x*_Mn_1–*x*_Cl_*y*_Br_3–*y*_@PMMA CFM with a thickness of 1 mm was stacked on a blue LED substrate and then sealed with black silicone to prevent light leakage and cured at room temperature for 30 min to obtain the WLED device. The emitted color can be adjusted by changing the thickness of the film.

### 2.3 Photocatalytic Experiment

The photocatalytic degradation experiment was conducted using a 250 mL solution of methyl orange (MO) with a concentration of 10 mg/L (pH ≈ 5.5). A 50 mg (contains approximately 1.50 μmol of perovskite, with the specific mass varying slightly depending on the doping ratio) sample of the electrospun fibrous membrane was fully immersed in the MO solution. The setup was kept in the dark for 30 min to allow for adsorption equilibrium. Following this, a magnetic stirrer was activated to ensure thorough mixing in the dark for an additional 30 min. Subsequently, a 300 W xenon lamp was used as the light source to initiate the photocatalytic reaction. The dye concentration was measured at 30 min intervals using a UV–vis spectrophotometer. The entire experimental process was carried out at room temperature under ambient conditions.

### 2.4 Characterization

The UV–visible absorption spectrophotometer was Shimadzu UV-2600 from Japan, the X-ray diffractometer (XRD) was D8 ADVANCE from Bruker Corporation, the contact angle measuring instrument was JC2000DS2B from Shanghai Zhongchen Digital Technology Co., Ltd., the scanning electron microscope (SEM) was Hitachi S-4800, the EDS was ZEISS Sigma 360/Carl Zeiss Microscopy Ltd., the electrospinning equipment was E03-001 from the R&D team of Foshan MBRT Nanofiber laboratories Technology Co., Ltd., and the photocatalytic instrument was an XPA series photochemical reactor from Xujiang Electromechanical Plant, Nanjing, China. The WLED was measured using the PCE-2000B single LED/mode photochromic electrical test system from EVERFINE. The system consists of a HASS-2000 high-precision fast spectral radiometer, LED300 test power supply, and integrating sphere for white LED testing.

## 3 Results and Discussion

To investigate the effect of different Mn doping levels on the properties of composite fiber membranes (CFM), we systematically varied the amount of MnCl_2_ in the precursor solution. MnCl_2_ was chosen as the doping agent based on previous research, which demonstrated that MnBr_2_ and MnI_2_ were less effective for doping due to bond energy limitations, making it challenging to introduce Mn into cesium lead halide perovskite.^29^ Earlier studies, such as those by Zhu et al., have successfully synthesized Mn-doped CsPbBr_3_ nanocrystals for light-emitting diode (LED) applications using MnCl_2_.^30^ However, Mn doping of CsPbBr_3_ membranes has not been explored previously. In this work, we prepared CFMs with different nominal doping concentrations, corresponding to Mn/Pb molar ratios of 0, 1:1, 2.5:1, and 5:1. The optical properties of the CFMs were examined under ultraviolet light, and the results are shown in the inset of Figure 1. The undoped CsPbBr_3_–PMMA membrane exhibits strong green luminescence, while the sample with a Mn:Pb ratio of 1:1 displays dark-blue luminescence. Further increasing the Mn doping concentration resulted in a progressive shift in luminescence color, from pink to purple red. This systematic change in emission color with Mn doping concentration highlights the tunable optical properties of the CFMs, which can be attributed to the incorporation of Mn into the perovskite structure and its influence on the electronic and photonic behavior of the material.

**Figure 1:**
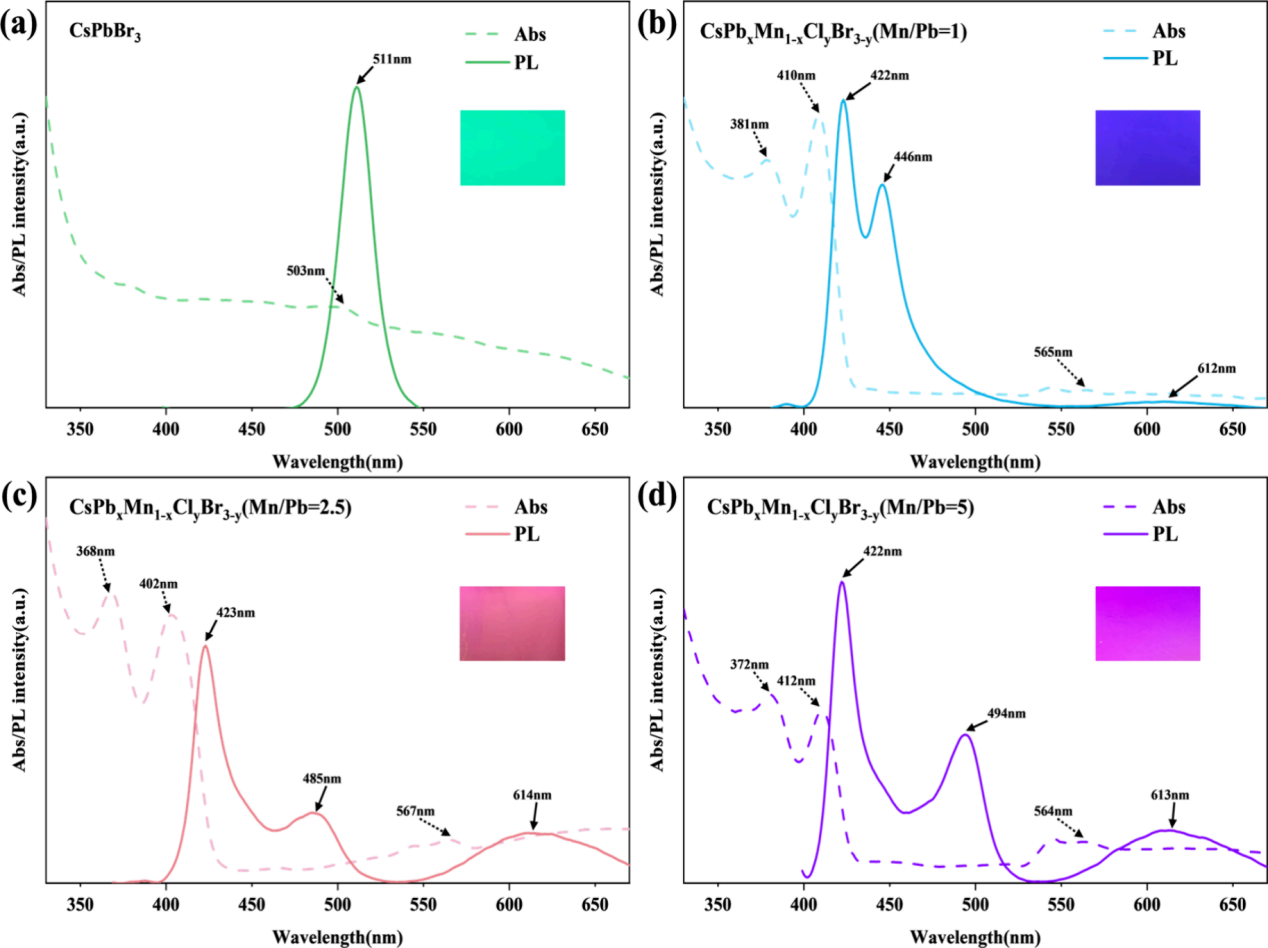
Photoluminescence spectra and UV absorption spectra of CsPb_*x*_Mn_1–*x*_Cl_*y*_Br_3–*y*_@PMMA CFMs with different Mn/Pb ratios: (a) Mn/Pb = 0; (b) Mn/Pb = 1; (c) Mn/Pb = 2.5; (d) Mn/Pb = 5. The insets show photos of samples under UV light.

Figure 1 illustrates the photoluminescence (PL) spectra of samples under a 350 nm excitation light. Without MnCl_2_ doping, the spectrum displays a single emission peak at 511 nm, corresponding to CsPbBr_3_. In line with the nomenclature introduced by Zhu et al., we denote our synthesized materials as CsPb_*x*_Mn_1–*x*_Cl_*y*_Br_3–*y*_ perovskite nanocrystals. Generally, as the Mn/Pb ratio increases, the *x* value decreases while the *y* value approaches 3, as previously demonstrated by Zhu et al. for nanoparticles. This results in two PL peaks (Figure.S1): one attributed to CsPb_*x*_Mn_1–*x*_Cl_3_ and another near 613 nm corresponding to the Mn^2+^ d–d emission (^4^T_1_ →^6^A_1_ transition).^30,31^ When nanocrystals are synthesized using the hot-injection method and ligand-assisted reprecipitation method, the resulting emission peaks are indeed consistent with those reported in the literature. However, in our CFM system, three PL peaks emerged upon MnCl_2_ doping. Similar results were obtained in repeated experiments, leading us to speculate that this phenomenon might be attributed to the influence of the electrospinning process on the material properties. This is different from the two peaks reported by Zhu et al. Consistent with Zhu’s findings, we observed an emission peak near 422 nm, which persisted across samples with increasing MnCl_2_ content, corresponding to the CsPb_*x*_Mn_1–*x*_Cl_3_ phase. Additionally, the 613 nm peak, associated with Mn^2+^ d–d emission, remained constant in position but increased in intensity with higher MnCl_2_ doping, explaining the shift in luminescence color to pink.^30^ A new emission peak appeared at 446, 485, and 494 nm for Mn/Pb ratios of 1, 2.5, and 5, respectively. These peaks correspond to a mixed CsPb_*x*_Mn_1–*x*_Cl_*y*_Br_3–*y*_ phase, where the *x* value decreases, and the Br content contribution increases relative to Cl. This behavior arises because, as the *y* value increases with MnCl_2_ doping, more Cl is consumed in forming CsPb_*x*_Mn_1–*x*_Cl_3_, leaving less Cl to participate in the mixed phase. The increasing Br contribution in the PL is corroborated by energy-dispersive X-ray spectroscopy (EDS) analysis, as detailed in Table S1. This observation of simultaneous PL peaks from the CsPb_*x*_Mn_1–*x*_Cl_3_ phase and Mn^2+^ d–d emission also aligns with findings by Fang et al. for nanocrystals.^32^ However, the emergence of a third PL peak in our CFM system highlights unique structural and compositional dynamics induced by Mn doping in the membrane architecture. Due to the multiple emission peaks and the competition among the multiple excited states and luminescent centers, PLQY was unable to differentiate between these dynamic processes and thus was not measured.

The impact of the Mn doping concentration on the crystal structure of CsPbBr_3_ was studied by X-ray diffraction (XRD). Figure 2 presents the XRD pattern of CsPb_*x*_Mn_1–*x*_Cl_*y*_Br_3–*y*_ with varying MnCl_2_ doping levels. The undoped sample exhibits a cubic phase of CsPbBr_3_, with its standard pattern indexed by PDF # 00-054-0752. The three main diffraction peaks at 2θ = 15.24, 21.78, and 30.67° correspond to the (100), (110), and (200) crystal planes of the cubic phase, respectively. Upon Mn doping, the XRD pattern shifts to reflect the tetragonal phase of CsPbCl_3_, indexed to PDF # 00-018-0366. The four main diffraction peaks at 2θ = 15.90, 22.52, 31.84, and 32.05° correspond to the (100), (101), (002), and (200) crystal planes of the tetragonal phase of CsPbCl_3_, respectively. This pattern did not align with the tetragonal phase of CsPbBr_3_, as the latter exhibits subtle differences in peak splitting at specific angles compared with its cubic phase. The transformation to the tetragonal phase of CsPbCl_3_ is attributed to the higher dissociation energies of Mn–Cl and Pb–Cl bonds compared to Mn–Br and Pb–Br bonds, favoring the formation of CsPbCl_3_ crystals through Mn^2+^, Pb^2+^, and Cl^–^ ions.^30^ Furthermore, the tetragonal structure is thermodynamically favored at lower temperatures.^33^ As the Mn doping level increases, the diffraction peak corresponding to the (200) crystal plane shifts to higher angles. According to Bragg’s law (2*d*sinθ = λ), a larger diffraction angle indicates a smaller lattice spacing (*d*). This shrinkage in lattice spacing is consistent with the substitution of Pb^2+^ (which is larger than Mn^2+^) by Mn^2+^ in the perovskite structure,^23^ confirming successful Mn doping. Furthermore, increased Mn doping leads to reduced crystallinity, potentially due to lattice defects or decreased particle size.^34^ Despite these changes, the crystal structure remains in the tetragonal phase of CsPbCl_3_.

**Figure 2:**
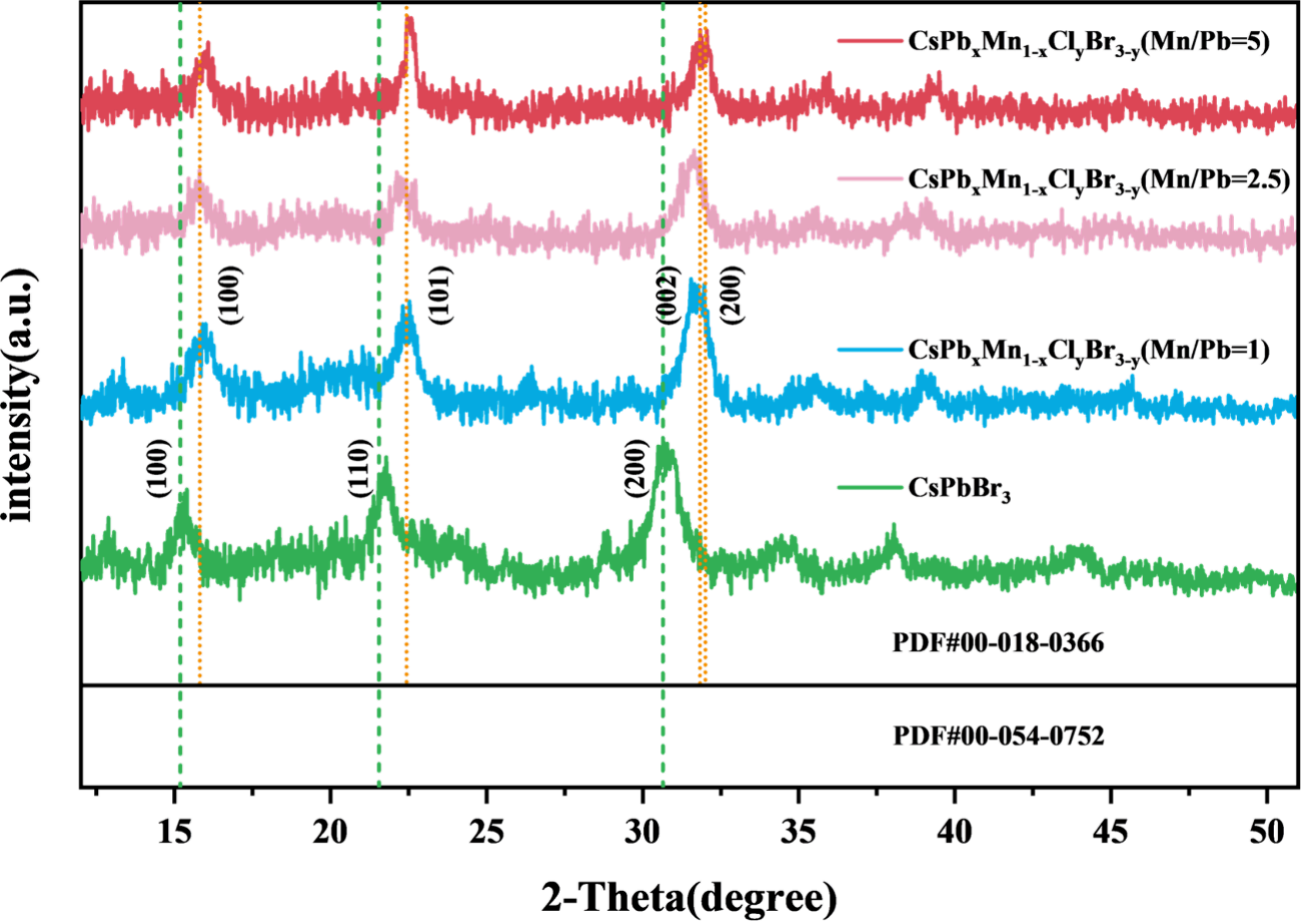
XRD diffraction pattern of CsPb_*x*_Mn_1–*x*_Cl_*y*_Br_3–*y*_@PMMA CFMs of Mn/Pb ratios 0, 1, 2.5, and 5, respectively. PDF#00-018-0366 represents tetragonal CsPbCl_3_ and PDF#00-054-0752 represents cubic CsPbBr_3_.

Figure 3 shows scanning electron microscopy (SEM) and energy-dispersive X-ray spectroscopy (EDS) images of CsPb_*x*_Mn_1–*x*_Cl_*y*_Br_3–*y*_@PMMA composite fiber membranes (CFMs). To determine the optimal PMMA concentration, experiments were conducted using PMMA concentrations of 25, 30, and 35 wt %, respectively. The SEM images of samples with 25 and 35 wt % PMMA are shown in Figure S2. It was found that at a PMMA concentration of 25 wt %, the fibers exhibited a disordered morphology with uneven thickness, and some fibers were curled. This was likely due to the low polymer concentration resulting in insufficient viscosity, making it impossible to form a uniform fiber structure under the electric field. When the concentration of PMMA was 30 wt %, as shown in Figure 3a,b, the CFM fibers exhibit a uniform morphology, forming an interconnected network without bead formation or aggregation. The fiber diameter ranges between 600 and 800 nm. When the concentration of PMMA was 35 wt %, the fibers were relatively uniformly distributed; however, their diameter increased significantly, with some even exceeding 4 μm, becoming excessively thick.

**Figure 3:**
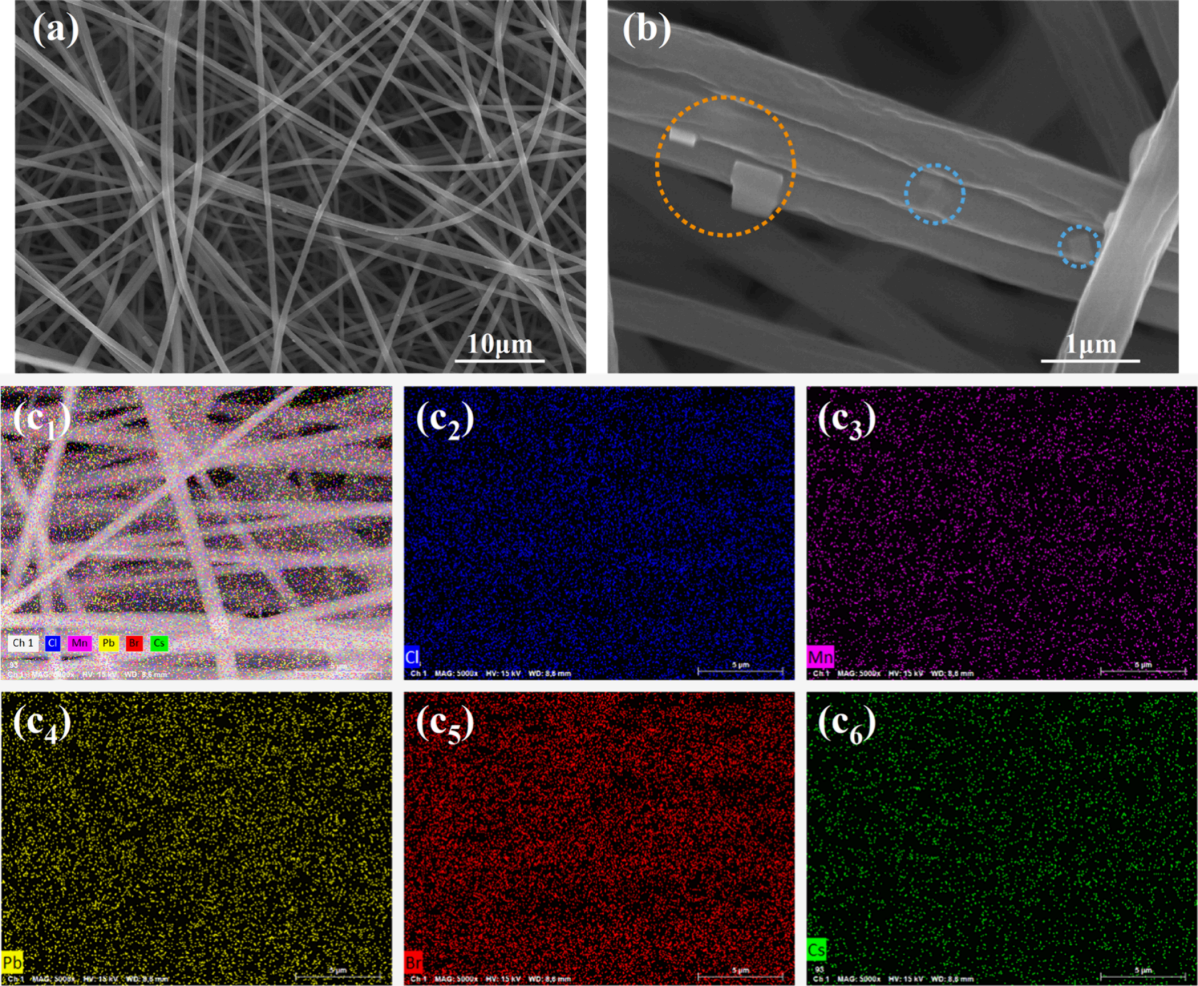
(a) and (b) show the SEM image of CsPb_*x*_Mn_1–*x*_Cl_*y*_Br_3–*y*_@PMMA CFMs for (Mn/Pb = 2.5). The orange circle in the figure represents the CsPb_*x*_Mn_1–*x*_Cl_*y*_Br_3–*y*_ nanocrystals on the surface of the fiber, and the blue circle represents the nanocrystals inside the fiber. (c_1_–c_6_) show the EDS elemental distribution map of CFM, (c_2_) Cl, (c_3_) Mn, (c_4_) Pb, (c_5_) Br, and (c_6_) Cs.

Furthermore, calculations reveal that for membranes of the same mass, the higher the PMMA concentration is, the lower the perovskite content. Taking a 50 mg membrane as an example, the molar content of perovskite is approximately 1.56 μmol when the PMMA concentration is 25 wt %, 1.50 μmol at 30 wt %, and 1.45 μmol at 35 wt %. The specific mass varies slightly with the doping ratio. Therefore, a PMMA concentration of 30 wt % was selected for the experiments, as it yielded membranes with the most uniform morphology and a relatively high perovskite content.

Aggregation of nanocrystals may lead to phase transitions, affecting their optoelectronic properties, and reducing stability.^33^ Electrospinning effectively prevents aggregation by distributing the CsPb_*x*_Mn_1–*x*_Cl_*y*_Br_3–*y*_ nanocrystals both on the surface and inside of the electrospun fibers. In Figure 3b, the orange circle highlights the CsPb_*x*_Mn_1–*x*_Cl_*y*_Br_3–*y*_ nanocrystals on the fiber surface, while the blue circle represents the nanocrystals inside the fiber. The surface nanocrystals are primarily involved in the catalytic process due to their direct contact with dye molecules. In contrast, the nanocrystals inside the fibers are protected, enhancing the photoluminescence (PL) properties of the CFM but potentially reducing photocatalytic performance.^18^ The EDS spectra in Figure 3 confirm the uniform distribution of Cs, Pb, Br, Cl, and Mn throughout the sample. Quantitative EDS data (Table S1) reveal that as the MnCl_2_ content increases, the *x* value of CsPb_*x*_Mn_1–*x*_Cl_*y*_Br_3–*y*_ decreases, while *y* increases and the Br content decreases.

The CFM demonstrates excellent hydrophobicity, with water contact angles ranging from 135 and 145° (Figure S3). This hydrophobic fibrous structure protects the perovskite nanocrystals, enabling photoluminescence in both air and water. As shown in Figure S4, all films maintain high PL emission in air over several weeks. Figure 4a–d illustrates that undoped and lightly doped CFMs retain good stability in water, ensuring reliable performance during 120 min photocatalytic tests. The disappearance of the Mn PL peak at 613 nm after 30 min in water suggests that the excited electrons from the ^4^T_1_ level react with water to generate ·OH or with dissolved oxygen to produce ·O_2_^–^.^35^ However, the deformation of the PL peak for CFM with Mn/Pb = 5 after 30 min in water indicates that excessive Mn doping may decrease crystallinity and increase defect density. SEM analysis (Figure S5) reveals that the CFMs with Mn/Pb = 5 exhibit numerous exposed CsPb_*x*_Mn_1–*x*_Cl_*y*_Br_3–*y*_ particles on the fiber surface compared to other samples. This exposure may increase reactivity with water, highlighting the importance of optimizing Mn doping levels to balance photocatalytic performance and structural stability.^3^ Overall, the CFM structure provides a robust platform for enhancing both the stability and the functionality of perovskite nanocrystals in practical applications.

**Figure 4:**
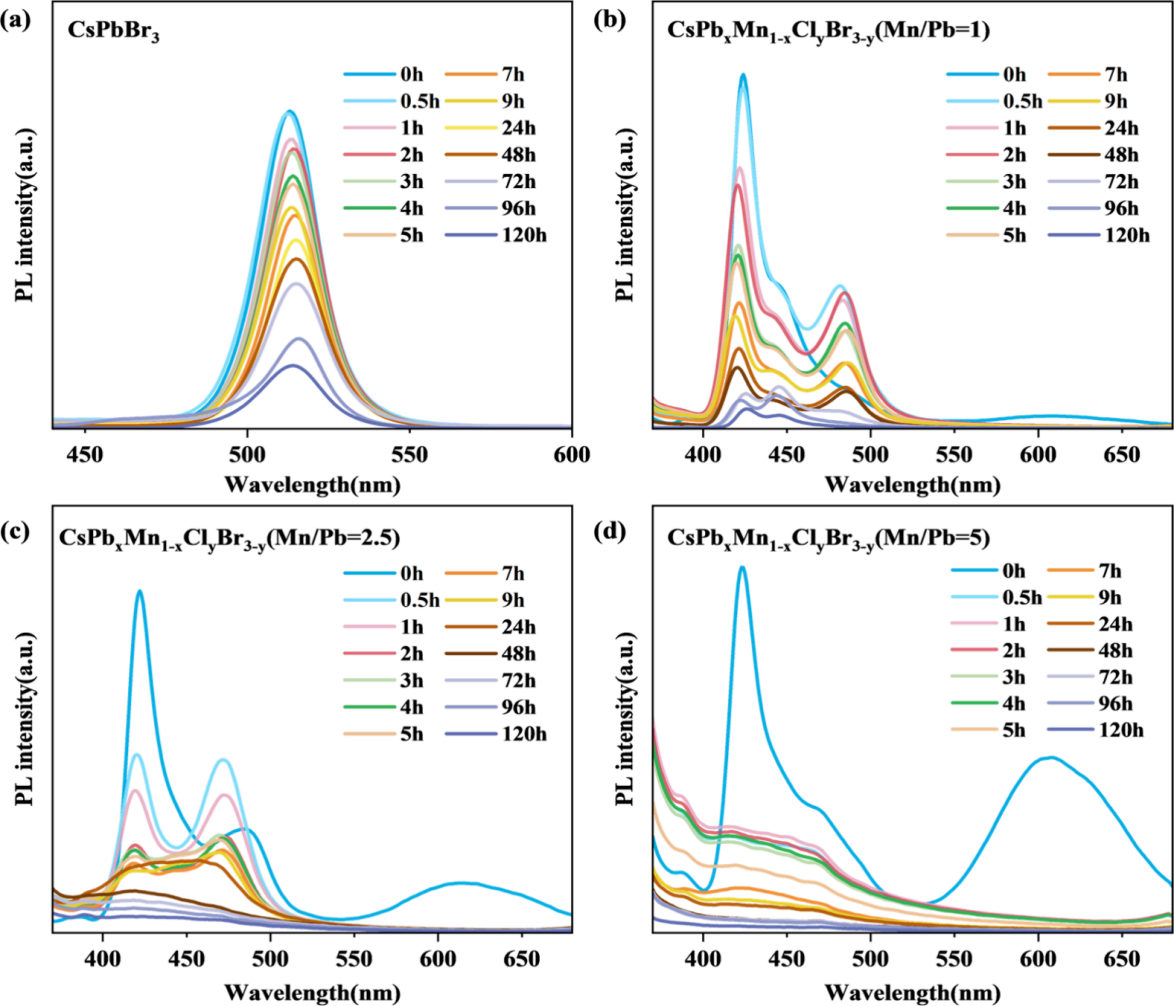
Stability of CsPb_*x*_Mn_1–*x*_Cl_*y*_Br_3–*y*_@PMMA CFMs in water for 120 h. (a) Mn/Pb = 0; (b) Mn/Pb = 1; (c) Mn/Pb = 2.5; (d) Mn/Pb = 5.

The photocatalytic performance of prepared CFM was evaluated using a 10 mg/L methyl orange (MO) solution, as shown in Figure 5. The excellent hydrophobicity of CFM protects the perovskite quantum dots in water while at the same time reducing the adsorption of the dye molecules, thereby maintaining the dye concentration during the adsorption experiment. For the undoped sample, there was almost no photocatalytic effect, and after 2 h of photocatalysis, the concentration of MO barely decreased. In contrast, the CFM based on CsPb_*x*_Mn_1–*x*_Cl_*y*_Br_3–*y*_@PMMA exhibited a significantly enhanced photocatalytic performance. The catalytic efficiency increased with Mn doping concentration, reaching its peak when the Mn/Pb ratio was 2.5. Specifically, after 1.5 h of photocatalysis, 94.73% of the original MO concentration was degraded, and after 2 h, the remaining concentration was only 2.11% of the initial value. This superior performance is attributed to the highest degradation efficiency and fastest degradation rate observed at this doping ratio, as confirmed by both the degradation efficiency and kinetics plots. The degradation kinetics of MO followed a pseudo-first-order model, described by −ln­(*C*_*t*_/*C*_0_) = *kt*, where *k* is the rate constant.^34^ However, further increasing the Mn concentration beyond Mn/Pb = 2.5 resulted in a decline in the photocatalytic performance. This is likely related to the reduced stability of the CFM in water, as evidenced by the water stability test, where the Mn/Pb = 5 sample exhibited the least stability among the doped samples. Despite this, its photocatalytic performance remained superior to that of the undoped sample, with the MO concentration reduced to 9.36% of the initial value after 2 h (Figure S6b,d). Notably, CFM prepared using MnCl_2_ alone (without CsPbBr_3_) demonstrated some photocatalytic activity due to the reaction of MnCl_2_ with water forming MnO_2_, which degrades dyes.^36^ However, this photocatalytic effect was much weaker than that of CsPb_*x*_Mn_1–*x*_Cl_*y*_Br_3–*y*_@PMMA. This suggests that while the surface MnCl_2_ residues may contribute to catalysis, the dominant photocatalytic performance originates from Mn^2+^ ions doped within the nanocrystals, consistent with prior studies on photocatalytic CO_2_ reduction.^16^ The optimal Mn doping ratio (Mn/Pb = 2.5) results from the combined effects of photocatalytic efficiency and stability, making it the most effective configuration for dye degradation.

**Figure 5:**
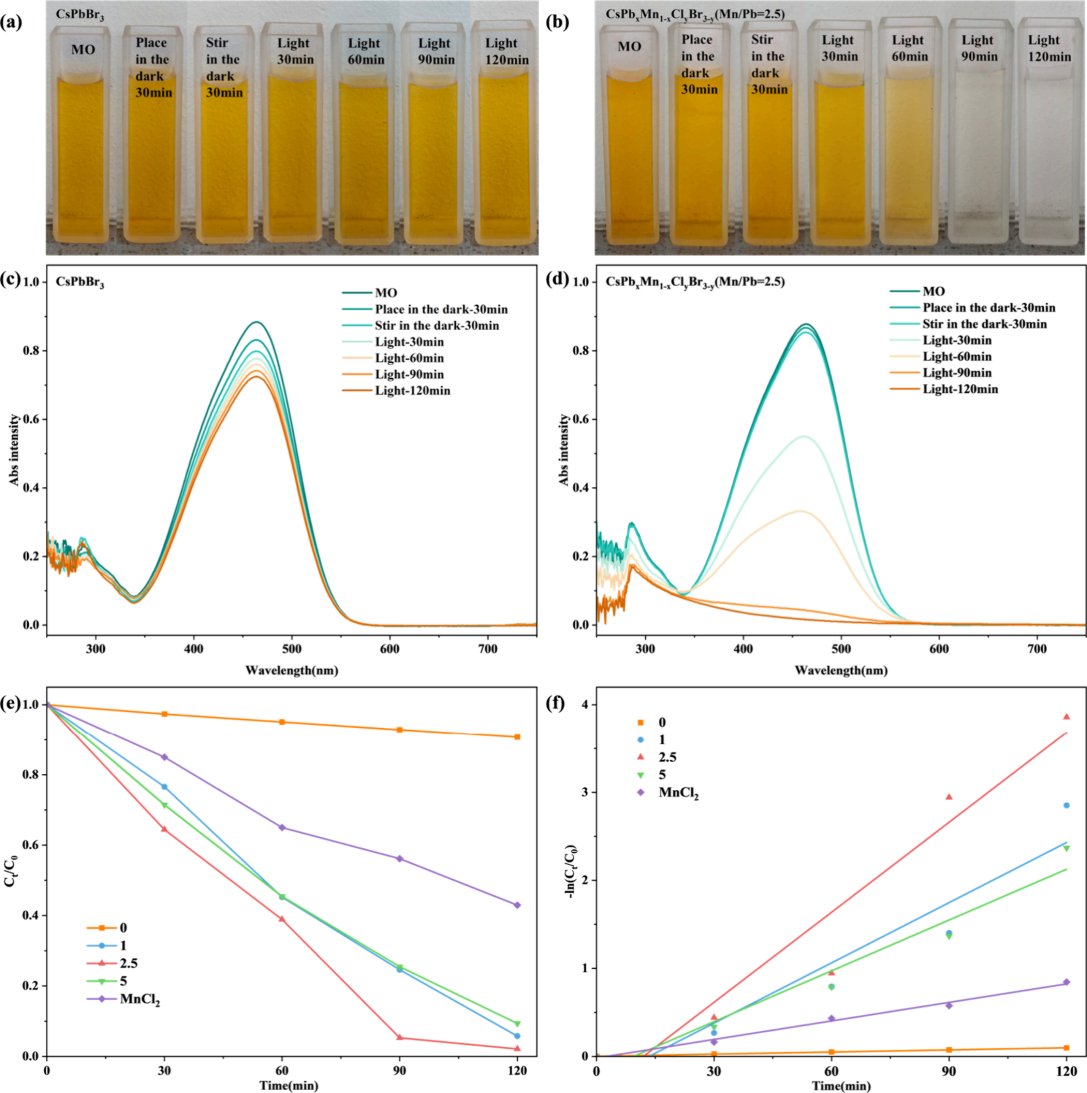
(a), (c), and (b), (d) are the photos and corresponding UV absorption spectra of the color change of methyl orange solution during the photocatalytic experiment for undoped samples and Mn/Pb ratio of 2.5, respectively. (e) Degradation efficiency and (f) degradation rate of methyl orange by photocatalysis for samples with different Mn/Pb ratios.

The PL emission and SEM images of the membranes after photocatalysis are shown in Figure S7. From the PL spectra, it can be observed that all membranes retained some PL emission after catalysis, although the intensity decreased. The SEM images reveal that the fibers of the postcatalysis membranes appear somewhat disordered and bent, with a less compact arrangement compared to their precatalysis state. However, the basic structure of the membranes remains intact. The retention of photoluminescence (PL) emission after photocatalysis indicates that the CFM maintains a certain degree of chemical stability, enabling it to complete the catalytic process without a complete degradation or loss of functionality. The stability of the CFM is attributed to the protective three-dimensional network structure of the electrospun fibers, which shields the internal CsPb_*x*_Mn_1–*x*_Cl_*y*_Br_3–*y*_ nanocrystals from direct interaction with water and light, thus preserving their PL properties. From the XRD test results, all Mn-doped samples exhibit characteristic peaks of tetragonal CsPbCl_3_, indicating that CsPbCl_3_ is the main phase of the material. It does not undergo significant changes with the variation of doping ratios and constitutes the major component of the material. Based on the photon energy formula *E*_g_ = *hc*/λ, where *hc* ≈ 1240 eV·nm, with the characteristic emission peak wavelength of 422 nm of CsPb_*x*_Mn_1–*x*_Cl_3_, it can be deduced that the band gap of CsPb_*x*_Mn_1–*x*_Cl_*y*_Br_3–*y*_@PMMA CFM is approximately 2.93 eV.

The photocatalytic mechanism of CsPb_*x*_Mn_1–*x*_Cl_*y*_Br_3–*y*_ nanocrystals is consistent with that of many photocatalytic materials, primarily involving the generation of superoxide radicals (·O_2_^–^) and hydroxyl radicals (·OH).^37,38^ Additionally, the photogenerated holes possess strong oxidizing capabilities and can directly oxidize water molecules to produce hydroxyl radicals (·OH).^37^ These highly reactive free radicals are the primary active species responsible for the degradation of methyl orange, breaking it down into nontoxic small molecules. The reaction mechanism is schematically illustrated in Figure 6 and summarized as follows:

$${\mathrm{CsPb}}_{x}{\mathrm{Mn}}_{1-x}{\mathrm{Cl}}_{y}{\mathrm{Br}}_{3-y}\mathrm{nanocrystals}+h\nu \to e^{-}+h^{+}$$

$$O_{2}+e^{-}\to •{O_{2}}^{-}$$

$$H_{2}O+e^{-}\to •\mathrm{OH}+{\mathrm{OH}}^{-}$$

$$H_{2}O/{\mathrm{OH}}^{-}+h^{+}\to •\mathrm{OH}$$

$$\mathrm{dye}+•\mathrm{OH}/•{O_{2}}^{-}\to \mathrm{intermediates}\to \mathrm{degradation}\;\mathrm{products}$$

**Figure 6:**
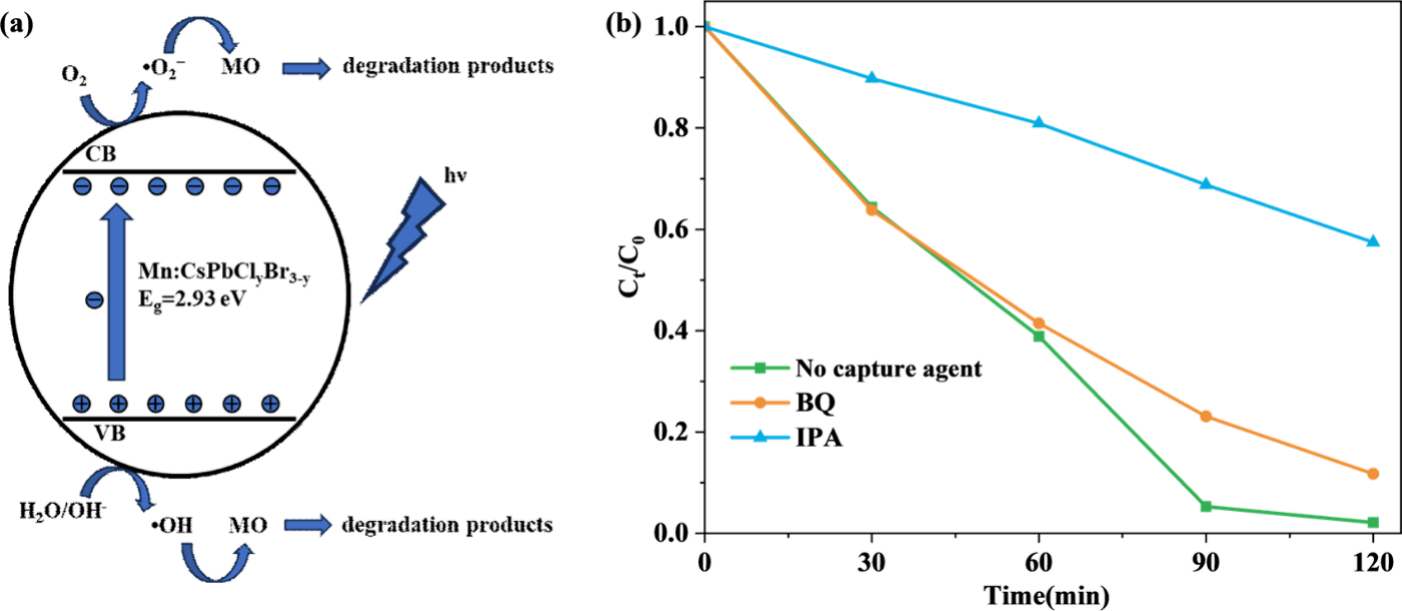
(a) Schematic diagram of the mechanism of CsPb_*x*_Mn_1–*x*_Cl_*y*_Br_3–*y*_@PMMA CFM photocatalytic degradation of methyl orange. (b) Comparison of degradation efficiency between adding capture agents and without.

To verify the role of hydroxyl and superoxide radicals in the catalytic experiment, trapping experiments were conducted for each case. The results are shown in Figure 6b. Isopropanol (IPA) was used to trap hydroxyl radicals, and *p*-benzoquinone (BQ) was employed to capture superoxide radicals. After adding IPA, the degradation efficiency decreased significantly, with only 42.57% of the original concentration degraded after 2 h. In contrast, the reduction in degradation efficiency was less pronounced after adding BQ, with 88.27% of the original concentration degraded within 2 h. These results indicate that hydroxyl radicals are the main active species, while the influence of superoxide radicals is relatively minor.

Due to the unique luminescent properties of CsPb_*x*_Mn_1–*x*_Cl_*y*_Br_3–*y*_@PMMA CFM, a WLED device was fabricated using a sample with a Mn/Pb doping ratio of 5, as it exhibits the highest Mn characteristic emission peak. First, CsPb_*x*_Mn_1–*x*_Cl_*y*_Br_3–*y*_@PMMA CFM of 1 mm thickness was stacked on a blue LED substrate, sealed with black silicone, and cured at room temperature for 30 min to obtain the WLED device. The structural diagram is shown in Figure 7a. The device was placed in the LED testing system and was evaluated with a current of 20 mA. The spectrum and physical image of the white LED are shown in Figure 7b, where distinct white light emission can be observed. The CIE color coordinates are shown in Figure 7c, with color coordinates (0.3267, 0.3298) very close to the standard white light coordinates, a color temperature of 5775 K, and a luminous efficiency of 132.33 lm/W. It was found to have an average rendition index (AvgR) of 81.3 and a color rendering index (CRI) of 85.66, reaching a good color rendering level Conventional cesium lead halide perovskite WLEDs typically require stacking of red and green films to achieve white light emission, whereas Mn-doped films can be accomplished with just a single monochromatic layer.

**Figure 7:**
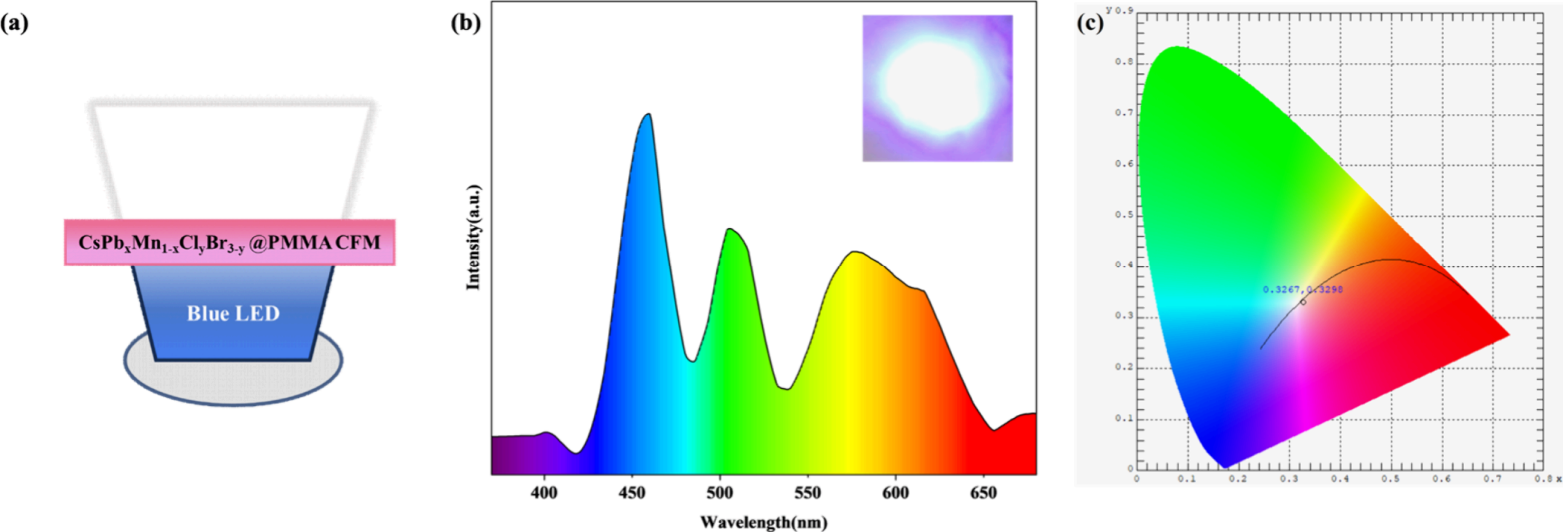
(a) WLED structure diagram prepared by CsPb_*x*_Mn_1–*x*_Cl_*y*_Br_3–*y*_@PMMA CFMs. (b) Spectral diagram of WLED (the inset is the physical picture of WLED). (c) CIE chromaticity coordinates of WLED.

To verify the stability of the LED, thermal stability and photostability tests were performed on the films, as shown in Figures S8 and S9. The thermal stability test revealed a certain correlation between the membrane’s emission and temperature: As the temperature increased, the exciton emission showed a downward trend (Figure S8e), whereas the Mn emission exhibited an opposite trend, rising continuously with increasing temperature (Figure S8f). For the photostability test, the membranes were irradiated with a 365 nm ultraviolet lamp at an intensity of approximately 15.3 mW/cm^2^. With an increasing irradiation time, the PL emission decreased. The samples with Mn/Pb ratios of 1 and 2.5 showed a significant red shift in their emission peaks after 1 day of testing. This may be due to the tendency of mixed halide perovskite membranes to undergo halogen migration under continuous light conditions, leading to segregation phenomena.^39,40^ The LEDs can be maintained in white light emission after 6 weeks at room temperature, with a color coordinate variation within (±0.77%, ± 5.28%), a color temperature variation within ± 1.73%, and an AvgR variation of ± 4.9%. Experiments have shown that although electrospun PMMA membranes help improve the stability of CsPb_*x*_Mn_1–*x*_Cl_*y*_Br_3–*y*_ nanocrystals, temperature and light can have a certain impact on the emission property of the membranes.

The prepared WLEDs were compared to Mn-doped or lead-free perovskite WLEDs. Fang et al. obtained results like ours in their temperature-dependence tests on Mn-doped CsPbCl_3_. They achieved in situ confined growth of perovskite nanocrystals within mesoporous silica spheres, which enhanced the stability and enabled the fabrication of WLEDs with excellent color rendering.^41^ The current lead-free perovskite WLED also made good progress. Zhu et al. fabricated WLEDs using Cs_3_Cu_2_X_5_ (X = Cl, Br) with a maximum color rendering index (CRI) of 98, which exhibit better thermal stability than traditional lead-based perovskites.^42^ In addition, they utilized 3D printing technology to encapsulate lead-free perovskites and other materials in resin separately. The resulting WLEDs feature favorable color coordinates and high color rendering indices. Resin protects the perovskite materials from environmental impacts, thereby enhancing the stability of both the color conversion layer and the entire WLED.^43^ In summary, encapsulation is an effective approach to protect perovskites and enhance their stability. In contrast, in our mixed halide perovskite membranes, it tends to undergo halogen migration under continuous light conditions, leading to segregation phenomena, which limits the thermal stability of the system.

Finally, to verify the feasibility of electrospun membranes for practical applications in the field of photocatalysis, a comparative analysis was conducted between these films and photocatalysts prepared by other methods. Compared with the sol–gel method, electrospinning technology uses polymers and metal salts as precursors, significantly reducing raw material costs.^44^ The sol–gel method typically relies on metal alkoxides as raw materials and requires large amounts of solvents and additives, leading to higher costs. Meanwhile, the high-temperature calcination process required by this method results in energy consumption much higher than that of electrospinning. In contrast to powder catalysts, membranes prepared by electrospinning not only address the issue of low recovery efficiency of powder catalysts^45,46^ but also have a specific surface area much larger than that of other materials, which can effectively reduce the amount of catalyst used.^44^ In addition, electrospinning also offers the advantage of a high production efficiency, making it more suitable for large-scale mass production. Electrospinning enables the large-area preparation of perovskite materials while maintaining their excellent performance, thus showing great potential in practical applications.^47^ Regarding WLEDs, although CsPb_*x*_Mn_1–*x*_Cl_*y*_Br_3–*y*_@PMMA CFM can achieve precise regulation of emission wavelengths through adjusting halogen ion ratios and introducing ion doping, thus demonstrating great potential as luminescent materials, they are prone to ion migration under the action of light and heat, which in turn triggers phase separation and causes changes in the emission peaks of the materials. Moreover, there is a competitive relationship between the emission from the introduced Mn ions and exciton emission. For instance, in thermal stability tests, the Mn emission peak and exciton emission peak show opposite changes with temperature variation. These are intrinsic limitations of mixed halide perovskite systems. Even with optimized preparation using electrospinning, their inherent characteristics restrict their practical application in WLEDs.

## 4 Conclusions

In this study, the CsPb_*x*_Mn_1–*x*_Cl_*y*_Br_3–*y*_@PMMA CFM was prepared by a one-step in situ electrospinning method. This approach offers several advantages, including enhanced stability of the perovskite nanocrystals by encapsulating them within the fibers, uniform dispersion of nanocrystals on the fiber surface to provide abundant catalytic sites, and ease of recovery without leaving residues in the treated water. Mn ion doping was found to significantly improve the photocatalytic performance of perovskite nanocrystals while reducing the content of Pb, addressing environmental concerns associated with Pb-based materials. The optimal doping concentration was determined to be Mn/Pb = 2.5, where the photocatalytic degradation of MO solution achieved exceptional efficiency, reducing the dye concentration to only 2.11% of its original value after 2 h of photocatalysis. The excellent photocatalytic performance, combined with the structural stability and environmental benefits, highlights the great potential of Mn-doped perovskite CFMs for practical photocatalytic applications in wastewater treatment and other environmental remediation processes. In addition, this CFM can be used to produce WLEDs with simple steps and good luminous performance. The chromaticity coordinates are (0.3267, 0.3298), and the color temperature is 5775 K.

## Supplementary Material



## Data Availability

More detailed data will be available upon request.

## Notes

The Supporting Information listed above is freely available online. The Supporting Information is available free of charge at https://pubs.acs.org/doi/10.1021/acsomega.5c06459.

(Figure S1) Fluorescence spectra of CsPb_*x*_Mn_1–*x*_Cl_*y*_Br_3–*y*_ nanocrystals with different Mn/Pb ratios prepared by ligand-assisted reprecipitation method; (Figure S2) SEM images of samples with PMMA concentrations of (a) 25 wt % and (b) 35 wt %; (Figure S3) water contact angle of CsPb_*x*_Mn_1–*x*_Cl_*y*_Br_3–*y*_@PMMA CFMs with different Mn/Pb ratios, (a) Mn/Pb = 0, (b) Mn/Pb = 1, (c) Mn/Pb = 2.5, and (d) Mn/Pb = 5; (Figure S4) stability of CsPb_*x*_Mn_1–*x*_Cl_*y*_Br_3–*y*_@PMMA CFMs in air for 8 weeks, (a) Mn/Pb = 0, (b) Mn/Pb = 1, (c) Mn/Pb = 2.5, and (d) Mn/Pb = 5; (Figure S5) SEM image of CsPb_*x*_Mn_1–*x*_Cl_*y*_Br_3–*y*_@PMMA CFM (Mn/Pb = 5); (Figure S6) photos of color change and the corresponding UV absorption spectra of methyl orange solution during the photocatalytic experiment for Mn/Pb ratio of 1 and 5, respectively; (Figure S7) PL spectra of CsPb_*x*_Mn_1–*x*_Cl_*y*_Br_3–*y*_@PMMA CFMs after photocatalysis for (a) Mn/Pb = 0, (b) Mn/Pb = 1, (c) Mn/Pb = 2.5, and (d) Mn/Pb = 5 and their SEM images for (e) Mn/Pb = 0, (f) Mn/Pb = 1, (g) Mn/Pb = 2.5, and (h) Mn/Pb = 5; (Figure S8) temperature-dependent PL spectra of CsPb_*x*_Mn_1–*x*_Cl_*y*_Br_3–*y*_@PMMA CFMs for (a) Mn/Pb = 0, (b) Mn/Pb = 1, (c) Mn/Pb = 2.5, and (d) Mn/Pb = 5 and (e) normalized intensity of exciton emission and (f) Mn^2+^ emission as a function of temperature; (Figure S9) stability of CsPb_*x*_Mn_1–*x*_Cl_*y*_Br_3–*y*_@PMMA CFMs under 365 nm ultraviolet light irradiation for 72 h for (a) Mn/Pb = 0, (b) Mn/Pb = 1, (c) Mn/Pb = 2.5, and (d) Mn/Pb = 5; and (Table S1) content of each element in CsPb_*x*_Mn_1–*x*_Cl_*y*_Br_3–*y*_@PMMA CFMs with different Mn/Pb ratios measured by EDS (PDF)

## References

[1] Xu L., Yuan S., Zeng H., Song J. (2019). A comprehensive review
of doping in perovskite nanocrystals/quantum dots: evolution of structure,
electronics, optics and light-emitting diodes. Mater. Today Nano.

[2] Protesescu L., Yakunin S., Bodnarchuk M. I., Krieg F., Caputo R., Hendon C. H., Yang R. X., Walsh A., Kovalenko M. V. (2015). Nanocrystals
of cesium lead halide perovskites (CsPbX3, X= Cl, Br, and I): novel
optoelectronic materials showing bright emission with wide color gamut. Nano Lett..

[3] Liu H., Wu Z., Shao J., Yao D., Gao H., Liu Y., Yu W., Zhang H., Yang B. (2017). CsPbxMn1-xCl3 Perovskite Quantum
Dots with High Mn Substitution Ratio. ACS Nano.

[4] Zhao Y., Xie C., Zhang X., Matras-Postolek K., Yang P. (2021). Mn: CsPbBr3 nanoplatelets
for bright white-emitting displays. ACS Applied
Nano Materials.

[5] Zhou Y., Liu C., Zhao Z., Zhang W., Li K., Ye Y., Zhu C. F., Meng X. G. (2020). Enhanced luminescence of Mn doped
CsPbCl3 and CsPb (Cl/Br) 3 perovskite nanocrystals stabilized in glasses. J. Alloys Compd..

[6] Yang Y., Liu X., Liu T., Chen D., Ye Z., Li J., Huang Q., Zhu Y., Pang Y., Zhang D., Liu Z., Cheng B., Zheng J., Zuo Y. (2023). High-Speed Broadband
Hybrid Perovskite Nanocrystals /Ge Photodetector from UV to NIR. Adv. Opt. Mater..

[7] Huang J., Wang H., Jia C., Tang Y., Yang H., Chen C., Gou K., Zhou Y., Zhang D., Liu S. (2024). Advances in crystallization
regulation and defect suppression strategies
for all-inorganic CsPbX3 perovskite solar sells. Prog. Mater. Sci..

[8] Che G., Wang X., Cui C., Pang B., Wang X., Dong H., Feng J., Yu L., Dong L. (2023). Boosting the
efficiency and stability of CsPbBr3 perovskite solar cells through
modified multi-step spin-coating method. J.
Alloys Compd..

[9] Liu W., Xie H., Guo X., Wang K., Yang C., Wang N., Ge C. (2023). Luminescence, stability, and applications
of CsPbBr3 quantum dot/polymethyl
methacrylate composites prepared by a solvent-and ligand-free ball
milling method. Opt. Mater..

[10] Zhang Q., Deng X., Tan C., Zhou Y., Chen X., Bai X., Li J., Tang B., Li S., Lin H. (2020). Gamma-phase
CsPbBr3 perovskite nanocrystals/polymethyl methacrylate electrospun
nanofibrous membranes with superior photo-catalytic property. J. Chem. Phys..

[11] Jiang M.-C., Pan C.-Y. (2022). Research on the
stability of luminescence of CsPbBr
3 and Mn: CsPbBr 3 PQDs in polar solution. RSC
Adv..

[12] Chen D., Zhou S., Tian F., Ke H., Jiang N., Wang S., Peng Y., Liu Y. (2019). Halogen-Hot-Injection
Synthesis of Mn-Doped CsPb­(Cl/Br)_3 Nanocrystals with Blue/Orange
Dual-Color Luminescence and High Photoluminescence Quantum Yield,
Advanced. Opt. Mater..

[13] Nuket P., Akaishi Y., Yoshimura G., Kida T., Vas-Umnuay P. (2022). situ TiO2-Coated
CsPbBr3 quantum dots with enhanced stability, photoluminescence quantum
yields, and charge transport properties. Ceram.
Int..

[14] Xu T., Xiahou J., Huang S., Liu Z., Li J. (2024). Dispersion
in water of CsPbBr3 perovskite nanocrystals by surface modification. Ceram. Int..

[15] Keerthana S., Yuvakkumar R., Ravi G., Al-Sehemi A. G., Velauthapillai D. (2022). Investigation
of optimum Mn dopant level on TiO2 for
dye degradation. Chemosphere.

[16] Wang J., Xiong L., Bai Y., Chen Z., Zheng Q., Shi Y., Zhang C., Jiang G., Li Z. (2022). Mn-Doped Perovskite
Nanocrystals for Photocatalytic CO_2 Reduction: Insight into the Role
of the Charge Carriers with Prolonged Lifetime. Solar RRL.

[17] Sudrajat H., Babel S., Ta A. T., Nguyen T. K. (2020). Mn-doped TiO2 photocatalysts:
Role, chemical identity, and local structure of dopant. J. Phys. Chem. Solids.

[18] Lv H., Liu Y., Bai Y., Shi H., Zhou W., Chen Y., Liu Y., Yu D. G. (2023). Recent Combinations
of Electrospinning with Photocatalytic
Technology for Treating Polluted Water. Catalysts.

[19] Zhang H., Mane A. U., Yang X., Xia Z., Barry E. F., Luo J., Wan Y., Elam J. W., Darling S. B. (2020). Visible-Light-Activated
Photocatalytic Films toward Self-Cleaning Membranes. Adv. Funct. Mater..

[20] Gomes G. H. M., de Jesus M. A. M. L., Ferlauto A. S., Viana M. M., Mohallem N. D. S. (2021). Characterization and application of niobium-doped titanium
dioxide thin films prepared by sol–gel process. Appl. Phys. A: Mater. Sci. Process..

[21] Kaneva N., Bojinova A., Papazova K. (2023). Enhanced removal of
organic dyes
using co-catalytic Ag-modified ZnO and TiO2 sol-gel photocatalysts. Catalysts.

[22] Zhang Q., Chen X., Wang H., Bai X., Deng X., Yao Q., Wang J., Tang B., Lin W., Li S. (2020). Controllable
synthesis of peapod-like TiO2@GO@C electrospun nanofiber membranes
with enhanced mechanical properties and photocatalytic degradation
abilities towards methylene blue. New J. Chem..

[23] Guria A. K., Dutta S. K., Adhikari S. D., Pradhan N. (2017). Doping Mn2+ in Lead
Halide Perovskite Nanocrystals: Successes and Challenges. ACS Energy Lett..

[24] Hou S., Gangishetty M. K., Quan Q., Congreve D. N. (2018). Efficient blue and
white perovskite light-emitting diodes via manganese doping. Joule.

[25] Chen D., Fang G., Chen X. (2017). Silica-coated Mn-doped CsPb (Cl/Br)
3 inorganic perovskite quantum dots: exciton-to-Mn energy transfer
and blue-excitable solid-state lighting. ACS
Appl. Mater. Interfaces.

[26] Shi W., Zhang X., Matras-Postolek K., Yang P. (2022). Mn-derived Cs 4 PbX
6 nanocrystals with stable and tunable wide luminescence for white
light-emitting diodes. Journal of Materials
Chemistry C.

[27] Huang Y., Pan Y., Guo S., Peng C., Lian H., Lin J. (2022). Large spectral
shift of Mn2+ emission due to the shrinkage of the crystalline host
lattice of the hexagonal CsCdCl3 crystals and phase transition. Inorg. Chem..

[28] Wang Q., Li K., Yang H., Lin D., Shih W. Y., Shih W.-H. (2022). Cesium
lead iodide electrospun fibrous membranes for white light-emitting
diodes. Nanotechnology.

[29] Yang Y., Li Q., Liu Y., Cong R., Sun Y., Hou J., Ge M., Shi J., Zhang F., Zhao G., Zhang N., Fang Y., Dai N. (2020). Magenta-Emitting Cesium Lead Halide
Nanocrystals Encapsulated in Dimethicone for White Light-Emitting
Diodes. ACS Applied Nano Materials.

[30] Zhu J., Yang X., Zhu Y., Wang Y., Cai J., Shen J., Sun L., Li C. (2017). Room-temperature synthesis
of Mn-doped cesium lead halide quantum dots with high Mn substitution
ratio. journal of physical chemistry letters.

[31] Ma K., Sheng Y., Wang G., Zhang X., Di Y., Liu C., Yu L., Dong L., Gan Z. (2022). Stable and multicolor
solid-state luminescence of Mn doped CsPb (Cl/Br) 3 perovskite quantum
dots and its application in light-emitting diodes. J. Lumin..

[32] Fang G., Chen D., Zhou S., Chen X., Lei L., Zhong J., Ji Z. (2018). Reverse synthesis of CsPb x Mn 1–
x (Cl/Br) 3 perovskite quantum dots from CsMnCl 3 precursors through
cation exchange. Journal of Materials Chemistry
C.

[33] Udayabhaskararao T., Kazes M., Houben L., Lin H., Oron D. (2017). Nucleation,
Growth, and Structural Transformations of Perovskite Nanocrystals. Chem. Mater..

[34] Chen P., Liang Y., Xu Y., Zhao Y., Song S. (2021). Synchronous
photosensitized degradation of methyl orange and methylene blue in
water by visible-light irradiation. J. Mol.
Liq..

[35] Wang J., Xiong L., Bai Y., Chen Z., Zheng Q., Shi Y., Zhang C., Jiang G., Li Z. (2022). Mn-Doped Perovskite
Nanocrystals for Photocatalytic CO2 Reduction: Insight into the Role
of the Charge Carriers with Prolonged Lifetime. Solar RRL.

[36] Chiam S.-L., Pung S.-Y., Yeoh F.-Y. (2020). Recent developments
in MnO2-based
photocatalysts for organic dye removal: a review. Environmental Science and Pollution Research.

[37] Gao G., Xi Q., Zhou H., Zhao Y., Wu C., Wang L., Guo P., Xu J. (2017). A Novel Inorganic Perovskite Quantum Dots for Photocatalysis. Nanoscale.

[38] Feng Y., Chen D., Niu M., Zhong Y., Ding H., Hu Y., Wu X., Yuan Z. (2023). Recent progress in metal halide perovskite-based
photocatalysts: physicochemical properties, synthetic strategies,
and solar-driven applications. Journal of Materials
Chemistry A.

[39] Guo Y., Yin X., Liu D., Liu J., Zhang C., Xie H., Yang Y., Que W. (2021). Photoinduced
self-healing of halide
segregation in mixed-halide perovskites. ACS
Energy Letters.

[40] Gao G. F., Chen Z. K., Lin K. S., Li Z. L., Gu H. T., Gao Y., Miao Y., Wu Y., Hu X., Polavarapu L. (2025). Unusual Nonlinear
Absorption Switching in Mixed-Halide Perovskites by Light-Induced
Halide Segregation. Laser Photon. Rev..

[41] Fang X., Zheng B., Ding M., Ye Y., Xu W., Han M., Lu J., Zhang B., Hong J., Huang H., Liu X., Wang J., Yuan Z. (2024). Light-induced synthesis of highly
stable CsPbCl3: Mn2+@ mSiO2 nanocomposites for white Light-Emitting
Diodes. Chem. Eng. J..

[42] Chen Y., Zhu H., Babaian D., Dzorkpata C., Grigoriev A., Wang Z., Wheat S., Guha S., Zhu P. (2025). Near-Unity
PLQY of CsCuX (X = Cl, Br) for High-Efficiency White Light-Emitting
Diodes with Exceptional Color Quality. Adv.
Mater..

[43] Yue Y., Zhu H., Dzorkpata C., Chen Y., Zhu P. (2025). High-performance
white
light-emitting diodes with stable 3-D-printed lead-free perovskite
color conversion layers. IEEE Trans. Electron
Devices.

[44] Samadi M., Moshfegh A. Z. (2022). Recent developments of electrospinning-based
photocatalysts
in degradation of organic pollutants: principles and strategies. ACS omega.

[45] Nguyen V. N. D., Leu H. J., Phan H. N. Q., Nguyen T. T., Ngo D. H. M. (2025). Investigating
the Recovery of PVDF/TiO 2 Photocatalyst for Methylene Blue Degradation. Processes.

[46] Wang P., Han X., Zheng X., Wang Z., Li C., Zhao Z. (2023). Removal of
Tetracycline Hydrochloride by Photocatalysis Using Electrospun PAN
Nanofibrous Membranes Coated with g-C3N4/Ti3C2/Ag3PO4. Molecules.

[47] Tian T., Yang M., Fang Y., Zhang S., Chen Y., Wang L., Wu W. Q. (2023). Large-area
waterproof and durable
perovskite luminescent textiles. Nat. Commun..

