# Supplementary material for: Manganese-Doped Cesium Lead Halide Perovskite PMMA Composite Fibrous Membranes by Electrospinning for Photocatalytic and LED Applications
Source: ACS Omega. 2025 Sep 25;10(39):45855–66. doi: 10.1021/acsomega.5c06459 (PMC12508993; doi:10.1021/acsomega.5c06459)
Supplement: Supplementary file 1 [file ao5c06459_si_001.pdf]

Supporting Information

Manganese doped cesium lead halide perovskite PMMA composite fibrous membranes by electrospinning for photocatalytic and LED applications

Yujie Cheng,[1] Yuang Ji,[1] Donghai Lin,[1*] Wan Y. Shih,[2] and Wei-Heng Shih[3*]

1 School of Energy and Materials, Shanghai Thermophysical Properties Big Data Professional Technical Service Platform, Shanghai Engineering Research Center of Advanced Thermal Functional Materials, Shanghai Key Laboratory of Engineering Materials Application and Evaluation, Shanghai Polytechnic University, Shanghai 201209, China.

2. School of Biomedical Engineering, Science, and Health Systems, Drexel University, USA

3. Department of Materials Science and Engineering, Drexel University, USA

*** Corresponding authors:**

Donghai Lin, dhlin@sspu.edu.cn; Wei-Heng Shih, shihwh@drexel.edu.

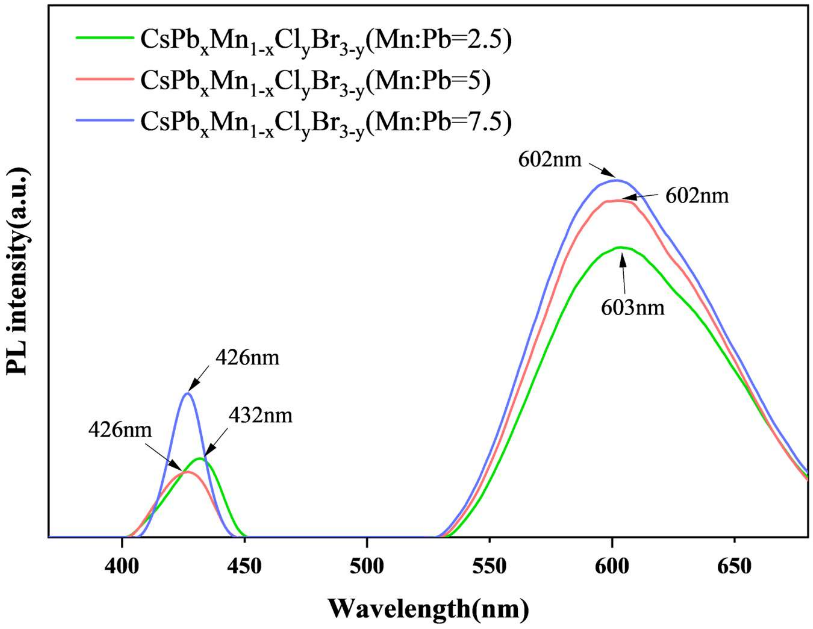

Fig. S1. Fluorescence spectra of $CsPb_xMn_{1-x}Cl_yBr_{3-y}$ nanocrystals with different Mn/Pb ratios prepared by ligand-assisted reprecipitation method.

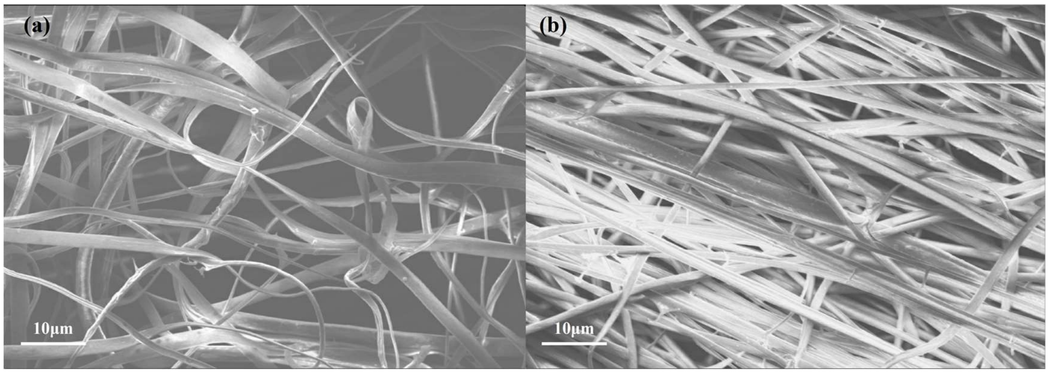

Fig. S2. SEM images of samples with PMMA concentrations of (a) 25 wt% and (b) 35 wt%.

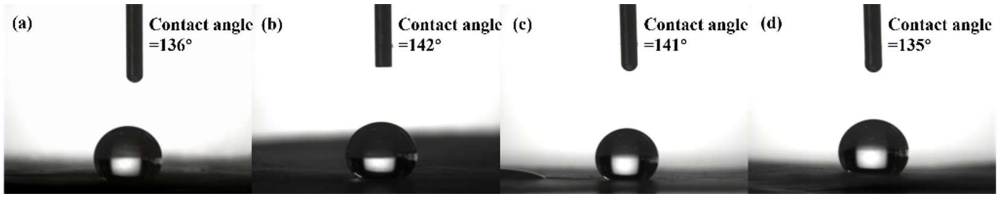

Fig. S3. The water contact angle of $CsPb_xMn_{1-x}Cl_yBr_{3-y}$@PMMA CFMs with different Mn/Pb ratios.(a) Mn/Pb=0; (b) Mn/Pb=1; (c) Mn/Pb=2.5; (d) Mn/Pb=5.

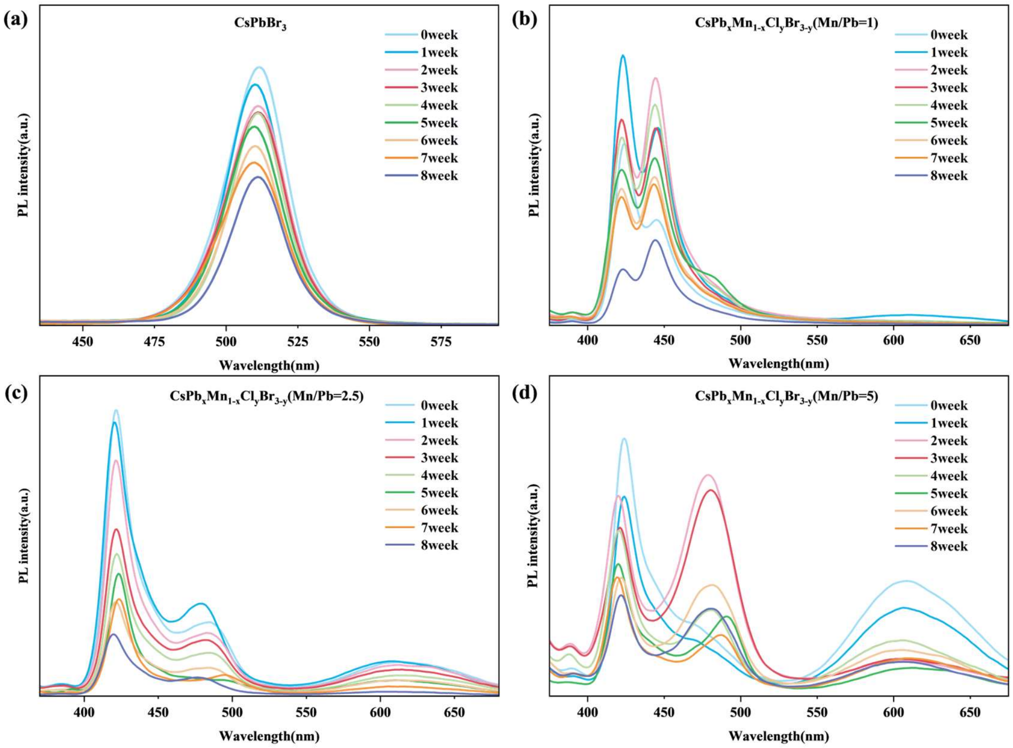

Fig. S4. Stability of $CsPb_xMn_{1-x}Cl_yBr_{3-y}$@PMMA CFMs in air for 8 weeks. (a) Mn/Pb=0; (b) Mn/Pb=1; (c) Mn/Pb=2.5; (d) Mn/Pb=5.

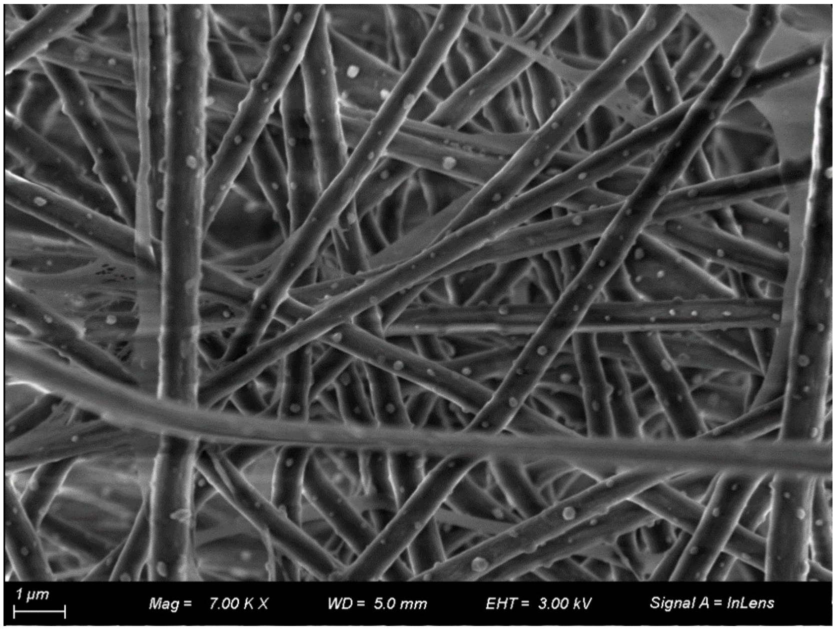

Fig. S5. SEM image of $CsPb_xMn_{1-x}Cl_yBr_{3-y}$@PMMA CFM (Mn/Pb=5).

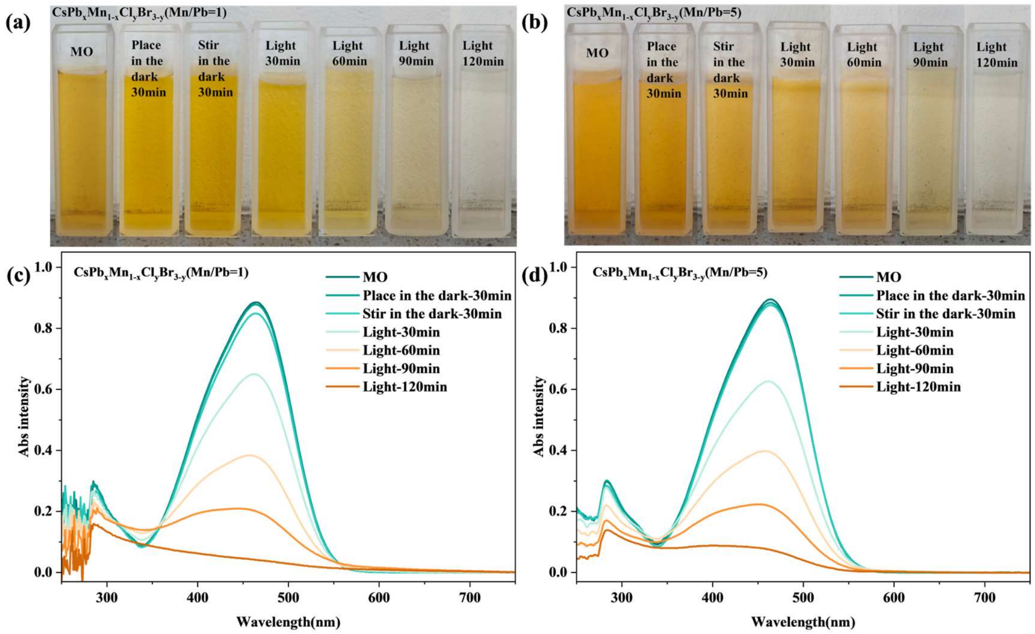

Fig. S6. (a) (c) and (b) (d) are the photos of color change and the corresponding UV absorption spectra of methyl orange solution during the photocatalytic experiment for Mn/Pb ratio of 1 and 5, respectively.

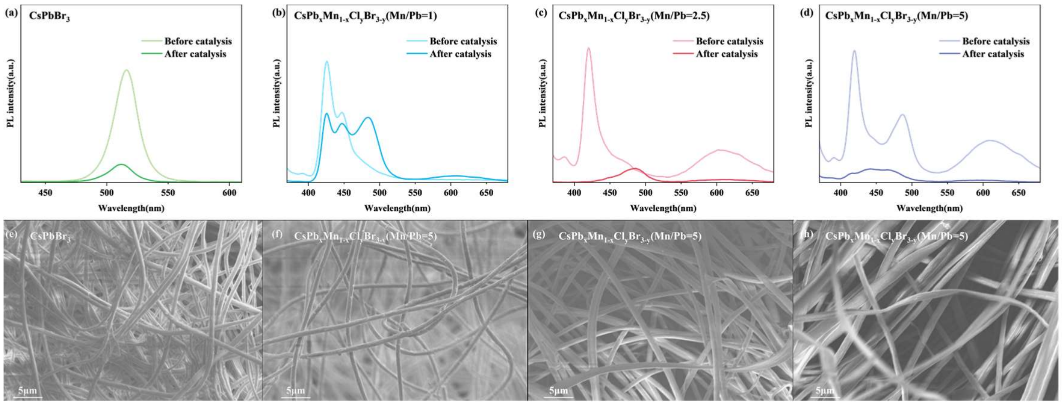

Fig. S7. PL spectra of $CsPb_xMn_{1-x}Cl_yBr_{3-y}$@PMMA CFMs after photocatalysis for (a) Mn/Pb=0; (b) Mn/Pb=1; (c) Mn/Pb=2.5; (d) Mn/Pb=5 and their SEM images for (e) Mn/Pb=0; (f) Mn/Pb=1; (g) Mn/Pb=2.5; (h) Mn/Pb=5.

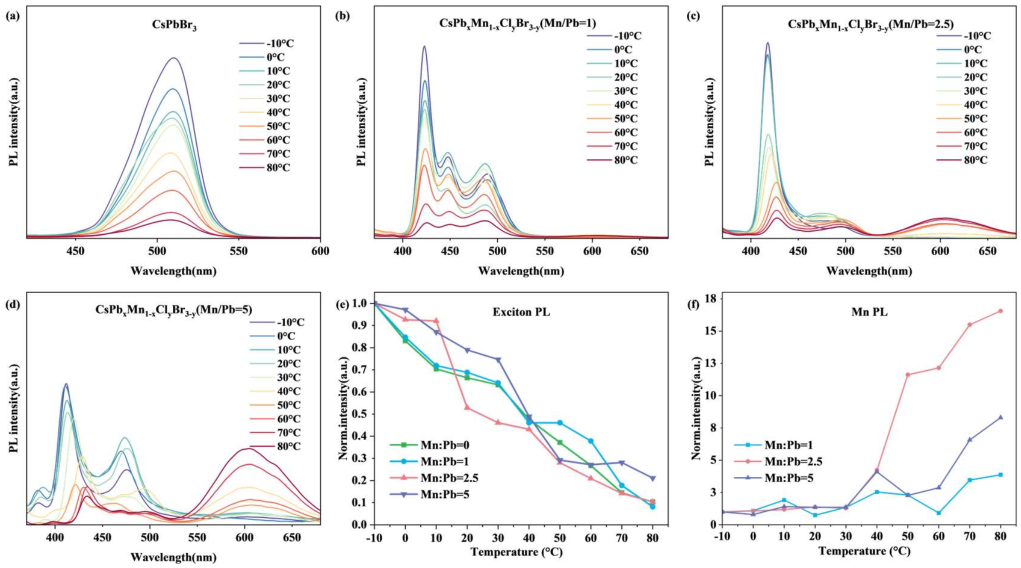

Fig. S8. Temperature dependent PL spectra of $CsPb_xMn_{1-x}Cl_yBr_{3-y}$@PMMA CFMs for (a) Mn/Pb=0; (b) Mn/Pb=1; (c) Mn/Pb=2.5; (d) Mn/Pb=5. (e) Normalized intensity of exciton emission and (f) Mn^{2+} emission as a function of temperature.

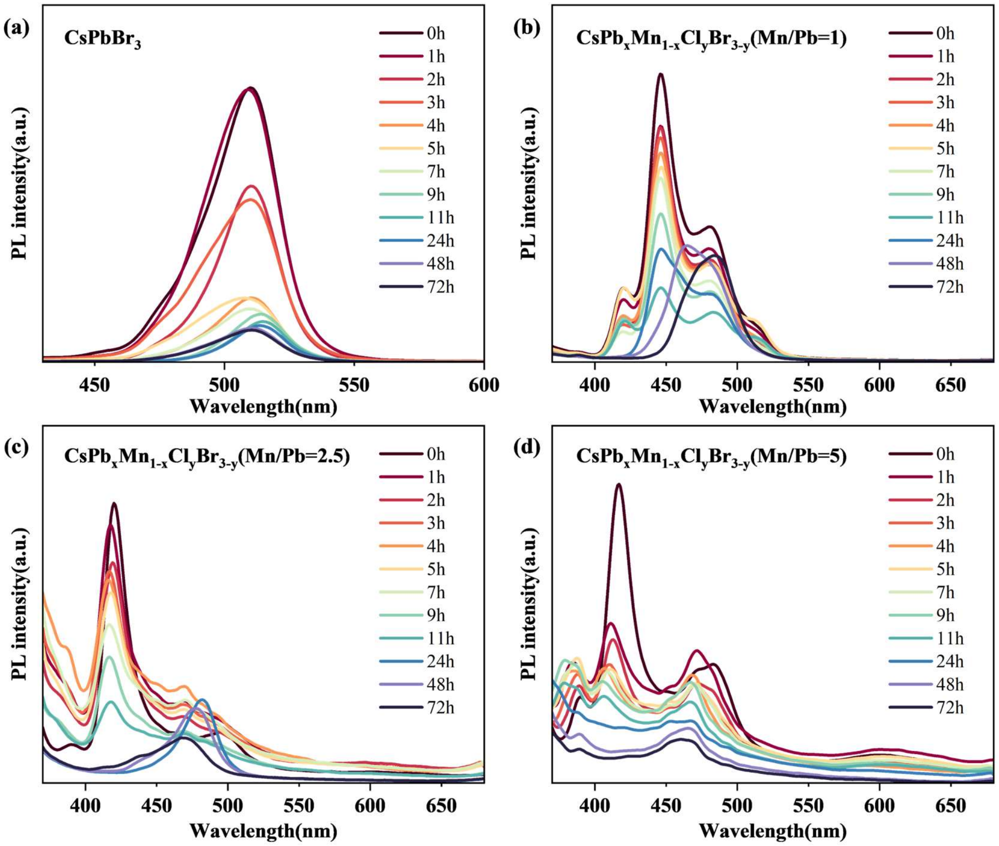

Fig. S9. The stability of $CsPb_xMn_{1-x}Cl_yBr_{3-y}$@PMMA CFMs under 365 nm ultraviolet light irradiation for 72 hours for (a) Mn/Pb=0; (b) Mn/Pb=1; (c) Mn/Pb=2.5; (d) Mn/Pb=5.

Mn/Pb feed ratio	Cs(%)	Pb(%)	Mn(%)	Cl(%)	Br(%)
1	8	22	5	16	49
2.5	9	17	8	26	40
5	6	14	8	33	39

Table.S1 Content of each element in $CsPb_xMn_{1-x}Cl_yBr_{3-y}$@PMMA CFMs with different Mn/Pb ratios measured by EDS.
